# Direct and Indirect Determinants of Body Mass Index in Both Major Ethnic Groups Experiencing the Nutritional Transition in Cameroon

**DOI:** 10.3390/ijerph19106108

**Published:** 2022-05-17

**Authors:** Emmanuel Cohen, Norbert Amougou, Amandine Ponty, Margaux Guerrien, Wakilongo Wakenge, Glory Chidumwa, Rihlat Said-Mohamed, Léopold K. Fezeu, Patrick Pasquet

**Affiliations:** 1 UMR7206 Eco-Anthropologie (EA), Muséum National d’Histoire Naturelle, CNRS, Université de Paris, Musée de l’Homme, 17 Place du Trocadéro, 75016 Paris, France; amandineponty@gmail.com (A.P.); margaux.guerrien@agroparistech.fr (M.G.); wwakilon@yahoo.fr (W.W.); patrick.pasquet@mnhn.fr (P.P.); 2 SAMRC/WITS Developmental Pathways for Health Research Unit, Department of Paediatrics, School of Clinical Medicine, Faculty of Health Sciences, University of Witwatersrand, 7 York Road, Parktown, Johannesburg 2193, South Africa; glorychidz@gmail.com (G.C.); rihlat.said-mohamed@wits.ac.za (R.S.-M.); 3 Department of Archaeology, Faculty of Human, Social and Political Science, School of Humanities and Social Sciences, University of Cambridge, Cambridge CB2 1TN, UK; 4 Sorbonne Paris Cité, Epidemiology and Statistics Research Center (CRESS), Inserm U1153, Inra U1125, Cnam, University of Paris 13, Nutritional Epidemiology Research Team (EREN), 93017 Bobigny, France; leopoldfezeu@icloud.com

**Keywords:** determinants, driver pathways, BMI, nutritional transition, Cameroon

## Abstract

In the context of rapid nutritional transitions in Africa, few studies have analyzed the etiology of obesity by considering the driver pathways that predict body mass index (BMI). The aim of this study is to innovatively identify these driver pathways, including the main sociodemographic and socioecological drivers of BMI. We conducted a rural–urban quantitative study in Cameroon (*n* = 1106; balanced sex ratio) to explore this issue. We recruited participants and reported several sociodemographic characteristics (e.g., marital status, socioeconomic status (SES), and ethnicity). We then assessed three main socioecological drivers of BMI (body weight perception, dietary intake, and physical activity) and conducted bioanthropometric measurements. We identified several driver pathways predicting BMI. In Cameroon, Bamiléké ethnicity, higher SES, being married, and older age had positive effects on BMI through overweight valorization and/or dietary intake. Accordingly, we found that being Bamiléké, married, and middle-aged, as well as having a higher SES, were factors that constituted at-risk subgroups overexposed to drivers of obesity. As such, this study highlights the necessity of investigating the complex driver pathways that lead to obesity. Therefore, better identification of the subgroups at risk for obesity will help in developing more targeted population health policies in countries where this burden is a major public health issue.

## 1. Introduction

The phenomenon of urbanization has already been experienced by Western countries through both massive rural exodus and the development of expanding built environments. While this urbanization is widely advanced in Asia, it is still ongoing in Africa. As such, the African continent currently presents one of the fastest urbanization rates in the world [1]. The urban environment is rapidly expanding in favor of greater access to motorized transports and processed energy-dense foods. Such a lifestyle transition leads to major nutritional and epidemiological changes through the rapidly increased prevalence of obesity associated with rising cardiometabolic non-communicable diseases (e.g., hypertension and type 2 diabetes) [2]. However, this obesogenic lifestyle transition is not homogenous in the continent, and some disparities in the progress of this phenomenon have appeared between countries. Some countries as South Africa and Cameroon present significantly higher rates of obesity, around 50% and 40% in urban adult women respectively [3,4], compared to those found in Senegal or Ghana, around 16% and 22% respectively [5,6].

The existing literature has identified the main socioecological (and behavioral) determinants of obesity related to the nutritional transition in Africa. Both physical and sociocultural environments can favor obesity. A greater access to processed energy-dense foods and motorized transports favors obesogenic dietary practices and sedentary behaviors [7,8], while the traditional social valorization of stoutness and idleness, as symbols of prosperity and peacefulness, leads to obesogenic fattening practices in urban environments [9]. However, the respective and combined contributions of these socioecological determinants remain insufficiently explored, and few studies include all of them in the same multivariable analyses [10]. Other studies generally consider only one, sometimes two, socioecological drivers together [5,11]. The respective and cumulative contributions of these socioecological drivers may yet explain the differences in prevalence and incidence of obesity between African countries.

In addition, these main socioecological determinants, related to the ongoing nutritional transition in Africa, do not equally expose individuals and populations according to their pre-existing sociodemographic characteristics. The sex, age, ethnicity, socioeconomic status (SES), etc., are (in absolute or short terms) themselves non-modifiable determinants of obesity [12,13]. However, in most studies, the multivariable analyses consider these determinants to be on the same level, as socioecological modifiable drivers stemming from the nutritional transition, especially in Africa [14,15]. Yet, the nature of these determinants is intrinsically different. This makes necessary to identify high-risk subgroups toward obesity, from their preexisting sociodemographic profile to their exposure levels to the main socio-ecological determinants of Body Mass index.

Indeed, several studies have stratified the determinants of obesity in order to identify specific pathways leading to weight gain. This stratification showed that these sociodemographic factors can be indirectly associated with BMI through physical activity and/or dietary intake, while the remaining factors can be directly associated with BMI [16,17,18]. Hence, the sociodemographic factors are commonly considered to be indirect (distal) determinants of BMI, and the socioecological factors within expanding urban environments are considered to be direct (proximal) determinants [19]. Thus, studying the indirect determinants of obesity has become a challenge for preventing this burden. There has been an increase in the number of studies analyzing these “determinants of determinants”—through body weight perception, dietary intake, and physical activity. Many of these studies were recently synthesized in several systematic reviews focusing on African women’s health [9,20,21]. 

Nevertheless, only very few studies in Africa have aimed to identify the pathways predicting obesity [22,23]. Urban obesogenic environments are rapidly expanding, involving differential biocultural adaptations that depend on population diversity in terms of gender, ethnicity, education, and other sociodemographic characteristics. Thus, in Cameroon, the exposure to urban environments does not equally affect the two main ethnic groups of the country: the Bamiléké and Béti. The obesity risk of the first group exceeds that of the second one by a factor three—a difference potentially caused by contrasting population eating habits and body size norms [24]. Structural equation modeling (SEM) is the appropriate statistical model to use for distinguishing direct and indirect associations between both sociodemographic and socioecological determinants, and BMI. Accordingly, this article aims to innovatively identify these direct and indirect driver pathways predicting the prevalence/incidence of obesity in Cameroon—including the main sociodemographic and socioecological drivers of BMI (Figure S1). This study will help to develop more targeted population health policies in Cameroon—a country where obesity has become a major public health issue—through the better identification of at-risk subgroups.

## 2. Materials and Methods

### 2.1. Study Design

We conducted a biocultural anthropological study among participants living in several villages (locally identified as Béti and Bamiléké) in rural Cameroon and in neighborhoods of Yaoundé [25]. Using this framework, we carried out a quantitative survey to assess the respective contribution of all BMI drivers investigated in this study. The survey included: (1) a sociodemographic questionnaire; (2) a validated African body image scale (Body Size Scales; BSS); (3) a food guide (Food Portion Photograph Book; FPPB); (4) a physical activity questionnaire (International Physical Activity Questionnaire; IPAQ); and (5) a bio-anthropometric protocol. Overall, the results from these integrated analyses facilitated the identification of direct and indirect driver pathways predicting obesity in Cameroon, including the main sociodemographic and socioecological drivers of BMI.

### 2.2. Sampling

In 2012, we used a two-stage sampling strategy to randomly select potential Bamiléké and Béti sites from an exhaustive list of Bamiléké and Béti villages. This method was also used to select sites in neighborhoods of Yaoundé. Hence, we recruited study participants from the following areas: two rural areas—one Bamiléké and one Béti, in West and Centre Cameroon, respectively; and two urban areas in Yaoundé—one mainly Bamiléké and one mainly Béti (1st degree). The participants covered a wide socioeconomic and education gradient according to Cameroon’s National Institute of Statistics. Subsequently, inside these respective clusters, for every three persons, we randomly selected one for inclusion (2nd degree). Inclusion criteria: age ≥ 18 years and self-identifying as Béti or Bamiléké. To collect reliable data, we excluded pregnant women and elders with reduced mobility.

### 2.3. Sociodemographic Characteristics 

Interviewer-administered questionnaires were used to collect sociodemographic information of participants. Marital status was categorized as married, cohabiting, single, divorced, or widowed. Educational level was categorized as a primary school or below, college, high school, and university. For each participant, place of residence was categorized as urban or rural, depending on whether they had lived in an urban area for at least 1 year. The migration profile of subjects was deduced through the creation of one variable, the “duration of residence in urban area”. This variable was coded into six categories: 0 years; 1–10 years; 11–20 years; 21–30 years; 31–40 years; and > 40 years. Duration of urban residence was investigated to see whether it was associated with nutritional status. SES was defined using the international wealth index, based on household assets described in the protocol of our previous Cameroonian study [25]. Participants were therefore classified into two categories: low SES and high SES. 

### 2.4. Perceptions of Corpulence 

To accurately assess body weight perceptions, we used the validated Body Size Scale (BSS) developed by our research team [26]. The BSS was treated as a metric value. Each human picture in the scale ranged from 1 to 9 according to increasing BMI categories. We constructed a body image assessment guide (BIAG) to contrast Bamiléké and Béti lay-person norms against the scientific body weight norms measured by the BSS. The BIAG consisted of three questions about the individual’s perceptions of current body size (CBS), desired body size (DBS), and ideal body size (IBS)—for oneself as well as one’s partner. The BIAG followed a standard protocol developed in our previous Cameroonian studies. It was used to assess possible differences in corpulence norms across geographical settings and between ethnic groups [3,24]. The fifth silhouette on the BSS corresponded to overweight; therefore, the social valorization of the overweight/obesity index was constructed as 4 minus DBS. 

### 2.5. Dietary Intake

The dietary intake of participants was determined using the Food Portion Photograph Book (FPPB). It was developed and validated for Central African populations by our research team to carry out culturally and culinary-relevant 24-h recall. The FPPB followed the protocol accurately described in our previous studies [25,27]. Therefore, the collected food data were used to assess the daily energy value in megajoules (MJ), calculated for each participant from portion sizes of food selected from the FPPB. 

### 2.6. Physical Activity

To assess the duration of physical activity, we used four items from the International Physical Activity Questionnaire (IPAQ) [28]. According to both ethnicity and rural–urban place of residence, the daily averages (in hours) of different activity levels were calculated from their respective durations in the seven days before completing the questionnaire. Activity levels were classified as: intense physical activity (digging, carrying heavy loads, etc.); moderate physical activity (carrying light loads, harvesting crops, cycling leisurely, etc.); light physical activity (walking, etc.) andsedentary behavior.

### 2.7. Anthropometry

A set of anthropometric measurements was taken by trained fieldworkers, using standardized procedures [29]. Height was measured to the nearest mm using a portable stadiometer (Siber Hegner, Zurich, Switzerland). Weight was measured with participants in very light clothing, to the nearest 100 g, using a digital beam scale (Tanita, Tokyo, Japan). Body mass index (BMI) was calculated by dividing weight (in kilograms) by the square of height (in meters). According to WHO recommendations, BMI (kg/m^2^) was categorized as underweight (<18.5); normal weight (18.5–24.9); overweight (25–29.9); and obese (≥30). Hip circumference (HC) and waist circumference (WC) were measured according to standard procedures, and the waist-to-hip ratio (WHR) was calculated to assess body fat distribution. Abdominal obesity was defined for WC as >88 cm in women and >102 cm in men; and for WHR as >0.85 in women and >0.90 in men [30]. Diastolic and systolic blood pressure (BP) measurements (mmHg) were taken with an electronic tensiometer (Omron France, Rosny-sous-Bois, France) after 15 min of rest, with the subjects in a seated position. Mean blood pressure was defined as diastolic BP + 1/3 * (systolic BP − diastolic BP) [31]. Hypertension was defined as diastolic BP ≥ 90 and/or systolic BP ≥ 140 mmHg. This biometric protocol allowed for assessment of the participants’ nutritional status and its association with hypertension. 

### 2.8. Data Analysis

We used Stata 14.1 software (StataCorp, College Station, TX, USA) for multiple linear regressions to assess anthropometric characteristics, body size standards, dietary intake, and physical activity. Chi-squared and Fisher’s exact test were conducted to assess the prevalence of overweight/obesity and hypertension. The relationships between the predictors (ethnicity, SES, education, marital status, age, urban residence duration, stoutness valorization (valorization of overweight), dietary intake, and physical activity) and BMI were assessed with generalized structural equation modeling (G-SEM) to identify direct and indirect driver pathways predicting this outcome. We conducted our analyses separately for males and females.

## 3. Results

### 3.1. General Characteristics of Participants

Participant characteristics are presented in Table 1. A total of 1106 participants were included in the present study. For Beti men, the mean age was 38 ± 14 years in urban area and 42 ± 14 years in rural area. In Bamiléké men, the mean age was 38 ± 14 years in urban area and 41 ± 14 years in rural area. The mean age of Béti women was 36 ± 13 years in urban area and 38 ± 13 years in rural area, whereas in Bamiléké women we found mean ages of 37 ± 13 and 39 ± 13 years in urban and rural areas, respectively. Concerning SES, regardless of gender, we found more participants with a high SES score in urban areas (Béti: 19 to 21%; Bamiléké: 18%) than in rural (Béti: 2 to 3%; Bamiléké: 2%). Regardless of gender and ethnicity, the proportion of married subjects was quite the same in both rural (14 to 16%) and urban (10 to 20%) areas. For both genders, we observed that the level of education was higher in urban (14 to 20%) than in rural (9 to 12%) areas, though Béti were more educated overall than Bamiléké for all areas and genders.

### 3.2. Perception of Corpulence

**Figure 1:**
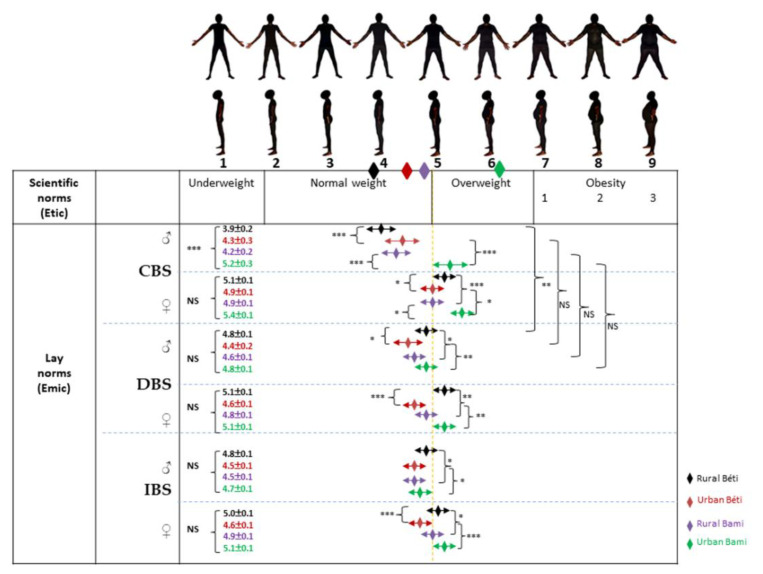
Perceptions of body size on masculine BSS. The diamonds just below the silhouettes correspond to the BMI averages of the four populations (rural Béti, urban Béti, rural Bami, and urban Bami). CBS: current body size; DBS: desired body size; IBS: ideal body size. ANCOVA between the four samples: * *p* < 0.05; ** *p* < 0.01; *** *p* < 0.001. Differences between CBS and DBS were tested using unpaired *t*-tests, * *p* < 0.05; ** *p* < 0.01; *** *p* < 0.001.

**Figure 2:**
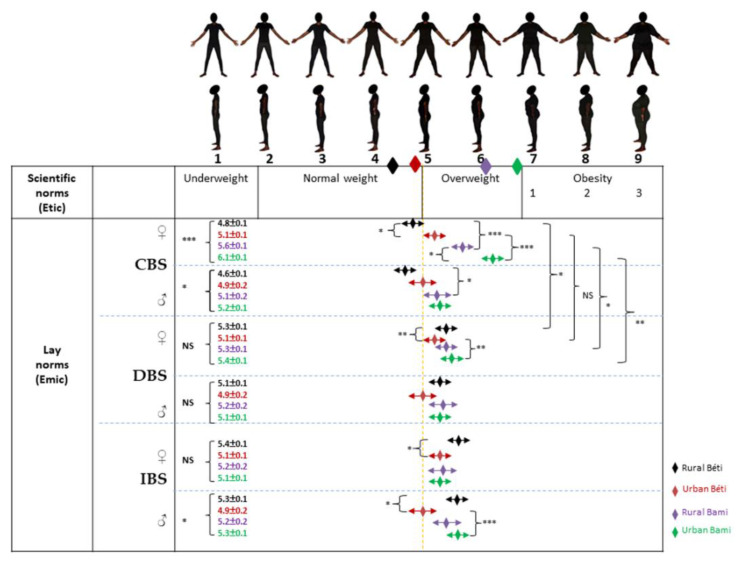
Perceptions of body size on feminine BSS. The diamonds just below the silhouettes correspond to the BMI averages of the four populations (rural Béti, urban Béti, rural Bami, and urban Bami). CBS: current body size; DBS: desired body size, IBS: ideal body size. ANCOVA between the four samples: * *p* < 0.05; ** *p* < 0.01; *** *p* < 0.001. Differences between CBS and DBS were tested using unpaired *t*-tests, * *p* < 0.05; ** *p* < 0.01; *** *p* < 0.001.

Perceptions of body size on masculine and feminine BSS are presented in Figure 1 and Figure 2, respectively. Among men, and for both Béti and Bamiléké, CBS means were significantly (*p* < 0.001) lower in the rural areas than in urban areas (Figure 1). In both genders, we observed that rural Béti valued overweight significantly more than their Bamiléké counterparts, while an opposite pattern was observed in the urban areas for both DBS and IBS means. In Béti only, we found that rural participants valued overweight significantly more than their urban counterparts for both DBS and IBS (except for IBS in men). Finally, CBS and DBS means were only significantly different for rural Béti, who desired to gain weight. 

Among Béti men, perceptions of themselves (CBS means) corresponded globally to their actual body size (BMI means), while a body size underestimation was observed in Bamiléké men. In the rural area, Béti women overestimated their partner’s body size. In the urban area, only Bamiléké women underestimated their partner’s body size. Men have shown DBS means in the normal weight range. However, we observed that women mainly wanted their partner to be overweight, except in urban Béti and rural Bamiléké. The IBS means for men were within the normal body size category, while women mainly idealized overweight for men, again with the exception of urban Béti and rural Bamiléké women.

Among women, and for both Béti and Bamiléké, CBS means were significantly (*p* < 0.001) lower in rural areas than in urban areas (Figure 2). We observed that urban Bamiléké valued overweight more than their Béti counterparts—in DBS for women, and IBS for men. In Béti only, we found that rural participants valued overweight significantly more than their urban counterparts in terms of DBS for women and IBS for both women and men. Finally, CBS and DBS means were significantly different in rural Béti and rural/urban Bamiléké; the rural Béti were willing to gain weight and the Bamiléké in both areas were willing to lose it. 

Among women, perceptions of themselves (CBS means) were overall oriented toward an overestimation in Béti and an underestimation in Bamiléké in terms of actual body size (BMI means). Only rural Béti men overestimated their partner’s body size, while a body size underestimation was observed in Bamiléké groups. Women have shown DBS and IBS means in the overweight category. However, we observed that only urban Béti men mainly wanted their partner (DBS) to be of normal weight and idealized the opposite sex (IBS) in this bodyweight category. The other groups had both DBS and IBS means in the overweight category.

### 3.3. Energy Value

In both genders and per ethnic group, we observed that the energy value was higher in the urban area than in the rural area. Overall, higher energy value means were found in Bamiléké compared to Béti, regardless of the place of residence or gender (Table 2). Regardless of gender, such ethnic differences in energy values were pronounced, such that even rural Bamiléké had higher energy value means (9.25 ± 0.23 to 9.69 ± 0.62 MJ) than those found in urban Béti (6.69 ± 0.14 to 8.57 ± 1.17 MJ) (Table 2).

### 3.4. Physical Activity

Regardless of gender, the average duration of intense physical activity was higher in the rural area than in the urban area. Overall higher intense physical activity was observed in Béti than in Bamiléké, per place of residence and for both genders (Table 2). In both genders and regardless of ethnicity (except for Bamiléké men), moderate physical activity was found to be higher in the urban area than the rural—although this physical activity was higher overall in Bamiléké than in Béti, regardless of gender (except for urban Béti women vs. Bamiléké men) (Table 2).

Regardless of gender, walking duration was longer in the rural area than the urban area, with an overall longer walking time for Bamiléké than Béti, per place of residence and for both genders. Per ethnic group, in men, sedentary activity was higher in urban dwellers than in rural ones, with a greater overall duration of sedentary activity observed in Bamiléké than in Béti. Only among Béti women, the tendency to be sedentary was higher in urban dwellers than in rural ones, with higher overall sedentary activity in Bamiléké than in Béti (Table 2). Regardless of gender, such ethnic differences in physical activities were pronounced, such that even rural Bamiléké had higher means for all physical activities (except for intense physical activity) than those found in urban Béti.

### 3.5. Bio-Anthropometry

Among men, a higher prevalence of overweight in the urban area was observed in both ethnic groups. We also observed a prevalence of obesity more than three times higher in urban Bamiléké compared to their rural counterparts, while obesity levels were similar, and low, in both rural and urban Béti (Table 3). Among women, a higher prevalence of overweight in urban area was only observed in Béti. On the other hand, we observed twice as much obesity in urban Bamiléké compared to their rural counterparts, while this prevalence was not much different in Béti. Per place of residence, we observed that the prevalence of overweight and obesity were significantly higher in Bamiléké than in Béti for both genders, except for overweight in urban women. Even the rural Bamiléké had higher prevalence of overweight and obesity than those found in urban Béti, except for overweight in men. Globally, similar trends were found for abdominal obesity. 

Per ethnic group, in both genders, mean blood pressure was lower in the rural area than in the urban area. In addition, again in both genders, the Bamiléké had overall higher blood pressure means than those found in Béti (Table 3). Therefore, even rural Bamiléké had higher blood pressure means than urban Béti (95.78 ± 1.49 vs. 92.96 ± 2.82 mmHg in men; 91.27 ± 1.13 vs. 90.34 ± 0.71 mmHg in women), with higher prevalence of hypertension in men (64 vs. 60%), and equal prevalence in women (42%).

### 3.6. Migration and Nutritional Status

**Figure 3:**
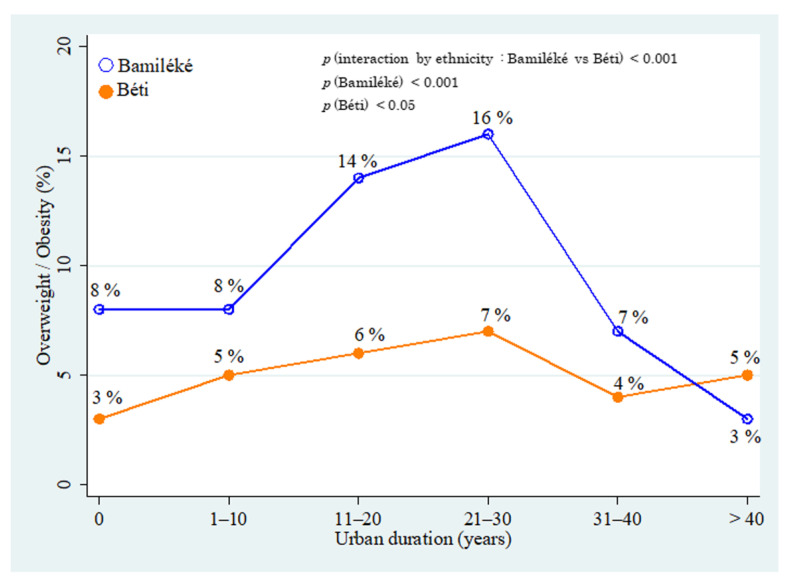
Association between the prevalence of overweight and migration status in Béti and Bamiléké, adjusted by age. The respective original value of each prevalence in Bamiléké was: 8% (*n* = 41); 8% (*n* = 47); 14% (*n* = 81); 16% (*n* = 95); 7% (*n* = 44); and 3% (*n* = 20). The respective original value of each prevalence in Béti was: 3% (*n* = 12); 5% (*n* = 26); 6% (*n* = 30); 7% (*n* = 40); 4% (*n* = 21); and 5% (*n* = 30). The *p* values to test the differences between Bamiléké and Béti (ethnicity), and the differences among Bamiléké and Béti are presented in the upper right.

Adjusted by age, we observed significant associations between overweight/obesity and the length of residence in the urban area for both Bamiléké (*p* < 0.001) and Béti (*p* < 0.05) (Figure 3). A difference between Bamiléké and Béti in the association between overweight/obesity and the length of residence in the urban area was also observed (*p* < 0.001). Before the starting conditions of urban residence (year 0), the Bamiléké showed a higher prevalence of overweight/obesity (8%) than Béti (3%). Between 1–10 years of urban residence, the prevalence of overweight/obesity was 5% in Béti and 8% in Bamiléké. At 10 years and at up to 30 years of urban residence, this prevalence marginally increased in Béti (from 6 to 7%) while it increased sharply in Bamiléké (from 14 to 16%). After 30 years of urban residence, we observed a pronounced decrease in both groups, with the effect of age adjustment, whilst the prevalence remained higher in Bamiléké (7 vs. 4%). Finally, after 30 years of urban residence, the prevalence of overweight/obesity were low and quite similar in both groups.

### 3.7. Driver Pathways Analysis

**Figure 4:**
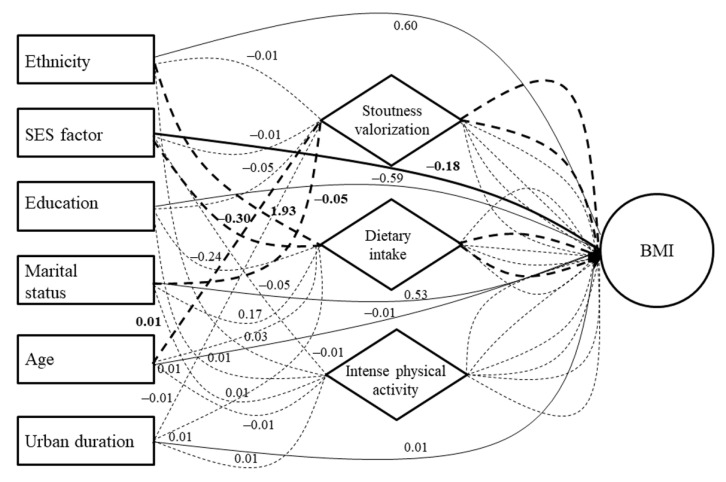
Results of structural equation modeling (SEM) showing associations between sociodemographic/socioecological predictors and body mass index in men. Continuous lines are direct sociodemographic effects and dotted lines are indirect sociodemographic effects. Bold lines (continuous and dotted) represent statistically significant paths. Simple lines (continuous and dotted) represent statistically non-significant paths.

Concerning the significant direct effects in men (Table S1), stoutness valorization and dietary intake predictors were positively associated with BMI, whereas the SES predictor (in an inverted direction) was negatively associated (Figure 4). Concerning the significant indirect effects in men (Table S2), the ethnicity predictor was found to be positively associated with BMI through dietary intake—whereas a negative BMI association was found for the SES predictor, again through dietary intake (Figure 4). The age predictor was then found to be positively associated with BMI through stoutness valorization, while a negative association was found for the marital status predictor, again through stoutness valorization.

**Figure 5:**
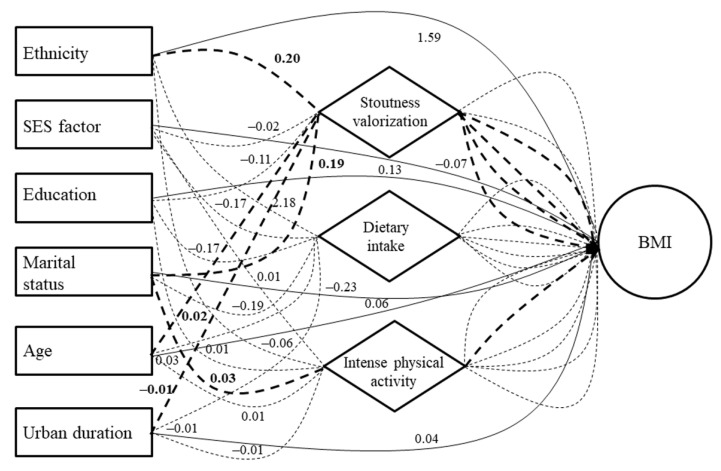
Results of structural equation modeling (SEM) showing associations between sociodemographic/socioecological predictors and body mass index in women. Continuous lines are direct sociodemographic effects and dotted lines are indirect sociodemographic effects. Bold lines (continuous and dotted) represent statistically significant paths. Simple lines (continuous and dotted) represent statistically non-significant paths.

Concerning the significant direct effects in women (Table S1), the stoutness valorization predictor was found to be positively associated with BMI (Figure 5). Concerning the significant indirect effects in women (Table S2), ethnicity, age, and marital status predictors were found to be positively associated with BMI through stoutness valorization. The last predictor was also positively associated with BMI through intense physical activity. Finally, the urban residence duration predictor was found to be positively associated with BMI, again through stoutness valorization (Figure 5).

## 4. Discussion

This study aimed to identify the different driver pathways leading to obesity in men and women, between six sociodemographic and three main socioecological obesogenic factors—social valorization of stoutness, energy-dense foods, and sedentary behaviors—stemming from the rapid nutritional transition underway in Cameroon. We first found that these socioecological factors were differently associated with the place of residence, ethnicity, and gender, demonstrating that the individual profile can influence the level of exposure to these drivers of obesity. Then, in line with previous results, we found association patterns between the place of residence and BMI. Finally, the SEM between all these variables showed several significant direct and indirect pathways predicting BMI in both genders. 

Concerning body weight perceptions, we observed that both Bamiléké and Béti women had body image means (DBS and IBS) in the overweight category—or at the frontier between normal weight and overweight for the opposite sex, in urban Béti. Bamiléké and Béti men presented body image means and standard deviations between normal weight and overweight categories. These trends are in accordance with previous African studies, such as those conducted in Nigeria and Senegal, showing that the valorization of stoutness is more associated with femininity, symbolizing fecundity and prosperity in the household [10,32]. Urban Béti men tended to value overweight less than their rural counterparts, while the urban Bamiléké men showed a propensity to value overweight considerably more than both their rural counterparts and urban Béti. Several studies in South Africa and Cameroon highlighted that this tendency in African cities to value overweight, decreased with the exposure to a modern urban lifestyle [23,33]. However, a case study of Bamiléké described their sociocultural norms and practices promoting stoutness as an identity marker of their agricultural and economic success in the rural–urban Cameroonian transition [24]. 

Concerning nutritional health aspects, the differences between genders for both prevalence of overweight/obesity (including abdominal obesity) are consistent with the existing literature, showing that women are more affected by the obesogenic effects of the nutritional transition [34,35]. In a study concerning rural–urban differences, Jones et al. [36] found similar tendencies in sub-Saharan Africa from the effect of the ongoing nutritional transition, where the prevalence of obesity and related cardiometabolic diseases increased with urbanization. Other studies in East and West Africa revealed similar trends through the rural–urban transition [2,37]. Finally, the ethnic differences observed between Bamiléké and Béti in their exposure to obesity risk can be explained by the propensity of the first group to maintain traditional habits that overvalue fatness and energy-dense culinary practices [24,38].

In this perspective, the rural–urban and ethnic differences found for the daily energy value can be similarly explained by two main effects: first, from the nutritional transition, favoring greater access to abundant, globally more processed, and energy-dense food, as observed in Ghana and South Africa [39,40]; and second, from the differential sociocultural food and body weight norms between Bamiléké and Béti [24]. In addition, both rural–urban and ethnic differences found for intense physical activity and sedentary behaviors followed precisely the same tendencies observed for body weight and dietary intake. This confirms that a nutritional transition is in progress in Cameroon, as observed in other African countries [25,35,37]—although, again, the starting conditions between individuals seem to have a strong ethnic component. Indeed, aside from the sociocultural traits detailed above, the Bamiléké from the rural area promote idleness in middle-aged people, viewing it as a symbol of prosperity, wellbeing, and successful integration into the urban lifestyle. This serves to exacerbate the obesogenic effects of the nutritional transition (i.e., favoring low physical activity levels in the rural area and lower physical activity levels in the urban area) [3]. 

The SEM showed that one socioecological driver, the valorization of stoutness, had a direct positive effect on BMI in both genders. This confirms the fact that the promotion of stoutness in Africa can be a risk factor for obesity, especially in the context of nutritional transition [5]. We also observed that higher SES and dietary intake in men were directly and positively associated with BMI. This difference between genders concerning these BMI drivers can be explained by the high prevalence of obesity in women. This suggests that for women, lifestyle conditions such as SES and eating practices have less effect on their BMI, unlike men being at an earlier stage in the nutritional transition in the urban area [34]. Indeed, the overexposure of women to obesity drivers, primarily favored by cultural factors that promote weight gain in women through fattening and idleness practices (β coefficient of stoutness valorization predictor: 2.53 in women vs. 0.5 in men), lead them to adopt significant and early obesogenic dietary patterns. This occurs within the transition process, erasing effects from other lifestyle conditions. On the opposite side, in men, recent SES improvements in the urban area can expose them to higher risks of obesity [41], as observed over several decades in low and middle income countries commonly situated at the early stages of the nutritional transition [2].

The SEM also showed several indirect driver pathways predicting BMI in our sample. In both genders, ethnicity was an indirect determinant that was positively associated with BMI through dietary intake in men and stoutness valorization in women. Indeed, the Bamiléké ethnic group tends to traditionally over-value stoutness and have a plentiful, energy-dense diet. They are recognized as such by other ethnic groups in Cameroon: a stout people who value fattening practices [24,42]. Subsequently, in both genders, we observed that age was an indirect determinant that was positively associated with BMI through stoutness valorization. This is consistent with the literature showing that African women and men in Cameroon, as well as in several other African countries, experience a social pressure to gain weight with age. This weight gain is viewed as a symbol of peacefulness in the household and is associated with fattening practices and higher prevalence of obesity at higher age ranges [9,10,24]. In addition, we observed in men that marital status was an indirect determinant negatively associated with BMI through stoutness valorization. This can be linked to the so-called “administrative belly” in Africa that represents a man’s success and prosperity, an expected criteria allowing easier access to marital life in traditional perceptions—weight gain being a symbol of responsibility and power for this gender [3,43]. 

We found that marital status was also positively associated with BMI through stoutness valorization in women. This confirms the fact that in African societies, women usually expect to gain weight after marriage as a symbol of household’s wellbeing [3,9]. We also observed that marital status was positively associated with BMI through intense physical activity. This can be explained by the fact that the overweight/obese married women in our sample had recently engaged in weight loss attempts, but had not yet had significant effects on their BMI, as observed in a South African study [4]. We then found that higher SES was positively associated with BMI through dietary intake. Such a trend is consistent with the literature showing a positive association between SES and BMI in populations at earlier stages of the nutritional transition, and who had recently improved their material lifestyle conditions, as observed in other African countries such as South Africa and Kenya [2,44]. Finally, we found that urban residence duration was negatively associated with BMI through stoutness valorization. This is in accordance with the cultural propensity in rural Cameroonian populations to value overweight, exposing them to overweight in the Bamiléké ethnic group [3,24].

The main strength of this work is the accurate analysis of the driver pathways that predict obesity in Cameroon in the context of a rapid nutritional transition. First, this approach is original in this region. Second, it can be used to better identify subgroups at-risk for obesity. Although the rapid urbanization process is global and concerns all African countries, the multiple ethnic groups and populations composing these nations have diverse historical backgrounds, anthropological characteristics, and sociodemographic profiles—articulated together, these features influence their adaptive capacities to evolve in emerging obesogenic urban environments [45]. This article innovatively included the prediction of BMI through complex driver pathways to provide a more accurate analysis on the etiology of obesity in Cameroon. This is despite the fact that the relatively small sample size assembled for this study may not provide the sufficient statistical power to obtain much in the way of significant results. We also did not explore any genetic factors of BMI, because this component is globally negligible compared to environmental ones [46], and no study of these ethnic two groups has attested to any kind of genetic differences that might influence BMI. In addition, the fact that we did not find any relevant associations between physical activity and BMI, especially with the metabolic equivalents tasks (METs) index of IPAQ, might be explained by our choice to include in our protocol a questionnaire to subjectively measure physical activity . This choice could be offset in the future through the use of its objective measurement with, for instance, accelerometry [47]. Finally, the delay of 10 years between data collection and data publication is explained by the fact that our research team could be trained on Stata software 14 to run SEM/GSEM in 2018. However, this does not affect the validity of the driver pathways of BMI that have been identified in this study, which are still in line with the Cameroonian society. 

Based on the main findings highlighted in this study on driver pathways predicting BMI in Cameroon, it appears that ethnicity, as well as other sociodemographic factors such as SES, marital status, and age, can shape the effects of the nutritional transition on the populations’ nutritional status and its main socioecological determinants. These last direct behavioral and modifiable determinants of obesity can be associated with BMI from indirect non-modifiable determinants related to population characteristics. Therefore, we recommend that international and national Cameroonian public health policies focus on the at-risk subgroups identified in this study to fight obesity. Indeed, from our main findings, public health authorities aiming to prevent and reduce obesity in Cameroon could develop more targeted interventions toward the Bamiléké ethnic group, along with married, middle-aged, and wealthier people, through the consideration of their specific norms, perceptions, and practices around body weight that can exacerbate the obesogenic effects of the nutritional transition within the recent and expanding built urban environment.

## 5. Conclusions

This study aimed to innovatively identify the direct and indirect driver pathways that predict BMI in Cameroon, including the main sociodemographic and socioecological determinants. The main findings showed that belonging to the Bamiléké ethnic group, being married, being middle-aged, and having a higher SES, constitute at-risk subgroups for obesity in the country. This study highlights the necessity of accurately investigating the direct and indirect driver pathways leading to obesity. The better identification of the subgroups at elevated risk for obesity will help to develop more targeted population health policies in the coming decades, especially in countries where this burden is a major or rising public health issue.

## Figures and Tables

**Table 1:** Characteristics of the studied population ^1^.

Ethnicity		Rural Béti	Rural Bamiléké	Urban Béti	Urban Bamiléké
Men	*n*	120	124	142	140
Age		42 ± 14 ^b-x^	41 ± 14 ^x-x^	38 ± 14 ^b-x^	38 ± 14 ^x-x^
SES (low) %		91 ^c-x^	93 ^c-x^	22 ^c-c^	12 ^c-c^
SES (high) %		9 ^c-x^	7 ^c-x^	78 ^c-c^	88 ^c-c^
Marital status %					
Married		53 ^c-x^	56 ^x-x^	37 ^c-c^	60 ^x-c^
Single/cohabiting		47 ^a-x^	44 ^x-x^	63 ^a-c^	40 ^x-c^
Educational level %					
None or Primary		43 ^c-c^	63 ^c-c^	18 ^c-c^	40 ^c-c^
High school/university		57 ^c-c^	37 ^c-c^	82 ^c-c^	60 ^c-c^
Women	*n*	110	130	164	176
Age		38 ± 13	39 ± 13	36 ± 13	37 ± 13
SES (low) %		91 ^c-x^	92 ^c-x^	27 ^c-x^	32 ^c-x^
SES (high) %		9 ^c-x^	8 ^c-x^	72 ^c-x^	68 ^c-x^
Marital status %					
Married		66 ^a-x^	64 ^x-x^	77 ^a-x^	62 ^x-x^
Single/cohabiting		34 ^a-x^	36 ^x-x^	23 ^a-b^	38 ^x-b^
Educational level %					
None or Primary		62 ^c-x^	63 ^c-x^	42 ^c-x^	48 ^c-x^
High school/university		38 ^c-x^	37 ^c-x^	58 ^c-x^	52 ^c-x^

^1^ Design and age-adjusted by linear regression analyses. Values for age are expressed as means ± SD (standard deviation) and were tested with linear regression models. Values for categorical variables are expressed as percentages (%) and were tested using chi-squared tests. a < 0.05; b < 0.01; c < 0.001; x = not significant (NS). The rural–urban ethnic comparisons followed this order: rural Béti vs. urban Béti (1st superscript in column 1 and 3); rural Bamiléké vs. urban Bamiléké (1st superscript in column 2 and 4); rural Béti vs. rural Bamiléké (2nd superscript in column 1 and 2); and urban Béti vs. urban Bamiléké (2nd superscript in column 3 and 4).

**Table 2:** Energy values from 24-h recall and physical activity means between Béti and Bamiléké in rural and urban areas ^1^.

Ethnicity		Rural Béti	Rural Bamiléké	Urban Béti	Urban Bamiléké
Men	*n*	120	126	143	140
Energy value (Mega Joule)		7.53 ± 0.94 ^x-b^	9.69 ± 0.62 ^a-b^	8.57 ± 1.17 ^x-c^	12.15 ± 0.92 ^a-c^
Intense physical activity (H)		2.28 ± 0.07 ^b-c^	1.23 ± 0.04 ^b-c^	1.03 ± 0.08 ^b-b^	0.40 ± 0.07 ^b-b^
Moderate physical activity (H)		1.50 ± 0.18 ^x-b^	2.07 ± 0.12 ^b-b^	1.66 ± 0.22 ^x-b^	1.81 ± 0.19 ^b-b^
Walking activity (H)		1.58 ± 0.12 ^a-c^	1.83 ± 0.08 ^a-c^	0.83 ± 0.15 ^a-c^	1.23 ± 0.12 ^a-c^
Sedentary behavior (H)		3.34 ± 0.15 ^b-c^	5.54 ± 0.10 ^c-c^	4.44 ± 0.19 ^b-c^	6.41 ± 0.15 ^c-c^
Women	*n*	110	132	164	179
Energy value (Mega Joule)		5.65 ± 0.01 ^b-c^	9.25 ± 0.23 ^c-c^	6.69 ± 0.14 ^b-c^	10.50 ± 0.11 ^c-c^
Intense physical activity (H)		1.31 ± 0.01 ^b-b^	0.91 ± 0.05 ^b-b^	0.29 ± 0.03 ^b-b^	0.14 ± 0.03 ^b-b^
Moderate physical activity (H)		1.59 ± 0.01 ^b-c^	2.33 ± 0.07 ^c-c^	1.82 ± 0.05 ^b-c^	2.39 ± 0.04 ^c-c^
Walking activity (H)		1.29 ± 0.01 ^b-c^	1.44 ± 0.07 ^c-c^	0.75 ± 0.05 ^b-b^	0.83 ± 0.04 ^c-b^
Sedentary behavior (H)		3.03 ± 0.01 ^a-c^	6.50 ± 0.20 ^a-c^	3.78 ± 0.12 ^a-c^	6.03 ± 0.09 ^a-c^

^1^ Design and age-adjusted by linear regression analyses. Values are expressed as means ± SD (standard deviation). H: hours. Linear regression models were used to test the differences between the four samples: a < 0.05; b < 0.01; c < 0.001; x = not significant (NS). The rural–urban ethnic comparisons followed this order: rural Béti vs. urban Béti (1st superscript in column 1 and 3); rural Bamiléké vs. urban Bamiléké (1st superscript in column 2 and 4); rural Béti vs. rural Bamiléké (2nd superscript in column 1 and 2); and urban Béti vs. urban Bamiléké (2nd superscript in column 3 and 4).

**Table 3:** Biometrics between Béti and Bamiléké in rural and urban areas ^1^.

Ethnicity		Rural Béti	Rural Bamiléké	Urban Béti	Urban Bamiléké
Comparative analysis for men	*n*	120	126	143	140
Body mass index (kg/m^2^)		21.80 ± 0.65 ^a-c^	24.21 ± 0.43 ^a-c^	23.46 ± 0.82 ^a-c^	26.76 ± 0.64 ^a-c^
Underweight (%)		10 ^c-c^	2 ^x-c^	5 ^c-c^	2 ^x-c^
Normal weight (%)		76 ^c-c^	66 ^c-c^	63 ^c-c^	37 ^c-c^
Overweight (%)		12 ^c-c^	26 ^c-c^	29 ^c-c^	40 ^c-c^
Obesity (%)		2 ^x-b^	6 ^c-b^	3 ^x-c^	21 ^c-c^
Waist circumference (cm)		77.12 ± 1.82 ^a-c^	84.18 ± 1.20 ^a-c^	80.52 ± 2.27 ^a-c^	89.97 ± 1.79 ^a-c^
No abdominal obesity		98 ^x-x^	96 ^c-x^	97 ^x-c^	84 ^c-c^
Abdominal obesity		2 ^x-x^	4 ^c-x^	3 ^x-c^	16 ^c-c^
Hip circumference (cm)		88.80 ± 1.30 ^a-b^	94.44 ± 0.86 ^a-b^	92.74 ± 1.62 ^a-b^	100.39 ± 1.28 ^a-b^
Waist-to-hip ratio		0.87 ± 0.01 ^x-a^	0.89 ± 0.01 ^x-a^	0.86 ± 0.01 ^x-a^	0.89 ± 0.01 ^x-a^
No abdominal obesity		99 ^x-x^	97 ^a-x^	98 ^x-a^	94 ^a-a^
Abdominal obesity		1 ^x-b^	3 ^c-b^	2 ^x-c^	6 ^c-c^
Mean blood pressure (mmHg)		91.42 ± 2.25 ^x-a^	95.78 ± 1.49 ^a-a^	92.96 ± 2.82 ^x-b^	100.23 ± 2.22 ^a-b^
No hypertension		53 ^a-c^	36 ^a-c^	40 ^a-c^	28 ^a-c^
Hypertension		47 ^c-c^	64 ^a-c^	60 ^c-c^	72 ^a-c^
Comparative analysis for women	*n*	110	132	164	179
Body mass index (kg/m^2^)		23.56 ± 0.03 ^c-c^	26.82 ± 0.46 ^b-c^	24.97 ± 0.26 ^c-c^	29.04 ± 0.19 ^b-c^
Underweight (%)		6 ^x-c^	0 ^x-c^	5 ^x-a^	2 ^x-a^
Normal weight (%)		65 ^c-c^	42 ^c-c^	53 ^c-c^	27 ^c-c^
Overweight (%)		18 ^c-c^	37 ^x-c^	28 ^c-x^	30 ^x-x^
Obesity (%)		11 ^a-c^	21 ^c-c^	14 ^a-c^	41 ^c-c^
Waist circumference (cm)		80.56 ± 0.12 ^a-c^	89.51 ± 1.28 ^x-c^	81.89 ± 0.72 ^a-c^	91.07 ± 0.53 ^x-c^
No abdominal obesity		81 ^c-c^	46 ^x-c^	67 ^c-c^	44 ^x-c^
Abdominal obesity		19 ^c-c^	54 ^x-c^	33 ^c-c^	56 ^x-c^
Hip circumference (cm)		94.07 ± 0.11 ^a-b^	103.04 ± 1.24 ^a-b^	98.15 ± 0.70 ^a-c^	108.09 ± 0.51 ^a-c^
Waist-to-hip ratio		0.86 ± 0.01 ^a-a^	0.87 ± 0.01 ^b-a^	0.83 ± 0.01 ^a-b^	0.84 ± 0.01 ^b-b^
No abdominal obesity		60 ^a-a^	51 ^a-a^	50 ^a-a^	40 ^a-a^
Abdominal obesity		40 ^a-a^	49 ^c-a^	50 ^a-b^	60 ^c-b^
Mean blood pressure (mmHg)		88.06 ± 0.03 ^a-a^	91.27 ± 1.13 ^a-a^	90.34 ± 0.71 ^a-b^	96.26 ± 0.53 ^a-b^
No hypertension		62 ^x-x^	58 ^c-x^	58 ^x-c^	39 ^c-c^
Hypertension		38 ^x-x^	42 ^c-x^	42 ^x-c^	61 ^c-c^

^1^ Design and age-adjusted by linear regression analyses. Values for continuous variables are expressed as means ± SD (Standard Deviation). Linear regression models were used for continuous variables. Values for categorical variables are expressed as percentages (%) and were tested by Chi-squared tests. a < 0.05; b < 0.01; c < 0.001; x = not significant (NS). The rural–urban ethnic comparison followed this order: rural Béti vs. urban Béti (1st superscript in column 1 and 3); rural Bamiléké vs. urban Bamiléké (1st superscript in column 2 and 4); rural Béti vs. rural Bamiléké (2nd superscript in column 1 and 2); and urban Béti vs. urban Bamiléké (2nd superscript in column 3 and 4). BMI: underweight (< 18.5 kg/m^2^); normal weight (18.5–24.9), overweight (25–29.9), and obese (≥ 30 kg/m^2^); Abdominal obesity: waist circumference > 88 cm (women), > 102 cm (men); WHR > 0.85 (women), > 0.90 (men); Hypertension: diastolic BP ≥ 90 and/or systolic BP ≥ 14.0.

## Data Availability

The authors do not want to publicly share the data related to this study.

## Supplementary Materials

The following supporting information can be downloaded at: https://www.mdpi.com/article/10.3390/ijerph19106108/s1, Figure S1: Conceptual framework of BMI driver pathways. Table S1: Results from structural equation models for all direct predictors and BMI. Table S2: Results from structural equation models for sociodemographic indirect predictors and BMI.
