# Supplementary material for: Direct and Indirect Determinants of Body Mass Index in Both Major Ethnic Groups Experiencing the Nutritional Transition in Cameroon
Source: Int J Environ Res Public Health. 2022 May 17;19(10):6108. doi: 10.3390/ijerph19106108 (PMC9141336; doi:10.3390/ijerph19106108)
Supplement: Supplementary file 1 [file ijerph-19-06108-s001.zip › Table S1.pdf]

Table S1. Results from structural equation models for all direct predictors and BMI.

	MALES		FEMALES	
**Stoutness Valorization**	**OR (95 % CI)**	***P*-Value**	**OR (95 % CI)**	***P*-Value**
Ethnicity				
Beti	Ref			
Bamileke	0.96 (0.84; 1.09)	0.269	1.14 (0.84; 1.57)	0.208
SES	0.92 (0.75; 1.12)	0.199	0.98 (0.91; 1.06)	0.420
Education				
Primary or less	Ref			
Above	0.59 (0.18; 1.97)	0.200	0.81 (0.31; 2.16)	0.459
Matrimonial status				
No married	Ref			
Married	0.75 (0.41; 1.35)	0.165	1.41 (1.10; 1.81)	**0.027**
Age	1.03 (1.00; 1.06)	**0.042**	1.04 (1.02; 1.05)	**0.014**
Urban duration	0.98 (0.94; 1.03)	0.202	1.00 (0.99; 1.01)	0.820
**Dietary intake (MJ)**	**β (95 % CI)**	***P*-value**	**β (95 % CI)**	***P*-value**
Ethnicity				
Beti	Ref			
Bamileke	707.3 (221.3; 1193.3)	**0.025**	1300.7 (659.9; 1941.4)	**0.013**
SES	-112.0 (-358.1; 134.8)	0.191	-97.9 (-277.1; 81.4)	0.143

Education				
Primary or less	Ref			
Above	-85.7 (-281.7; 110.4)	0.201	-96.0 (-993.4; 801.5)	0.691
Matrimonial status				
No married	Ref			
Married	74.2 (-240.5; 388.9)	0.417	-120.8 (-795.3; 553.8)	0.552
Age	6.2 (-34.0; 46.4)	0.576	16.3 (1.41; 32.2)	**0.042**
Urban duration	4.7 (-10.9; 20.3)	0.325	-2.46 (-28.6; 23.6)	0.724
**Intense physical activity (hours)**	**β (95 % CI)**	***P*-value**	**β (95 % CI)**	***P*-value**
Ethnicity				
Beti	Ref			
Bamileke	-0.86 (-1.58; -0.15)	**0.035**	-0.38 (-1.57; 0.81)	0.305
SES	0.25 (-0.15; 0.65)	0.116	0.05 (-0.06; 0.17)	0.194
Education				
Primary or less	Ref			
Above	0.03 (-0.44; 0.50)	0.806	-0.01 (-0.67; 0.66)	0.961
Matrimonial status				
No married	Ref			
Married	-0.18 (-0.8; 0.44)	0.342	0.20 (-0.19; 0.59)	0.157
Age	0.01 (-0.01; 0.03)	0.134	0.01 (-0.01; 0.03)	0.153
Urban duration	-0.01 (-0.04; 0.02)	0.273	-0.02 (-0.05; 0.01)	0.091

BMI (kg/m²)	β (95 % CI)	*P*-value	β (95 % CI)	*P*-value
Dietary intake	0.01 (0.01; 0.01)	**0.002**	0.01 (-0.01; 0.01)	0.274
Stoutness valorization	0.50 (0.22; 0.79)	**0.017**	2.53 (0.54; 4.52)	**0.032**
Intense physical activity	-0.04 (-0.24; 0.16)	0.478	0.20 (-0.81; 1.22)	0.483
Ethnicity				
Beti	Ref			
Bamileke	0.60 (-0.72; 1.92)	0.190	1.59 (-5.30; 8.47)	0.426
SES	-0.18 (-0.31; -0.06)	**0.024**	-0.07 (-0.58; 0.44)	0.613
Education				
Primary or less	Ref			
Above	-0.59 (-1.41; 0.23)	0.091	0.13 (-3.77; 4.04)	0.898
Matrimonial status				
No married	Ref			
Married	0.53 (-0.1; 1.15)	0.069	-0.23 (-2.46; 2.01)	0.707
Age	-0.01 (-0.05; 0.05)	0.931	0.06 (-0.01; 0.14)	0.071
Urban duration	0.01 (-0.01; 0.03)	0.248	0.04 (-0.07; 0.15)	0.242

SES: Socioeconomic status, coded in an inverted direction.
