# Supplementary material for: Direct and Indirect Determinants of Body Mass Index in Both Major Ethnic Groups Experiencing the Nutritional Transition in Cameroon
Source: Int J Environ Res Public Health. 2022 May 17;19(10):6108. doi: 10.3390/ijerph19106108 (PMC9141336; doi:10.3390/ijerph19106108)
Supplement: Supplementary file 1 [file ijerph-19-06108-s001.zip › Table S2.pdf]

Table S2. Results from structural equation models for sociodemographic indirect predictors and BMI.

MALES			FEMALES	
	**β (95 % CI)**	***P*-value**	**β (95 % CI)**	***P*-value**
**Ethnicity**				
Via Intense physical activity	0.03 (-0.03; 0.09)	0.288	-0.06 (-0.18; 0.06)	0.307
Via Stoutness valorization	-0.01 (-0.04; 0.02)	0.424	0.20 (0.09; 0.30)	**0.000**
Via Dietary intake	1.93 (1.60; 2.26)	**0.000**	2.18 (-0.13; 4.49)	0.065
**SES**				
Via Intense physical activity	-0.01 (-0.02; 0.01)	0.129	0.01 (-0.01; 0.01)	0.438
Via Stoutness valorization	-0.01 (-0.02; 0.01)	0.061	-0.02 (-0.07; 0.04)	0.576
Via Dietary intake	-0.30 (-0.59; -0.01)	**0.048**	-0.17 (-0.38; 0.05)	0.122
**Education**				
Via Intense physical activity	-0.01 (-0.02; 0.02)	0.876	0.01 (-0.08; 1.00)	0.855
Via Stoutness valorization	-0.05 (-0.14; 0.04)	0.272	-0.11 (-0.30; 0.09)	0.278
Via Dietary intake	-0.24 (-0.63; 0.16)	0.243	-0.17 (-0.76; 0.41)	0.561
**Matrimonial status**				
Via Intense physical activity	0.01 (-0.01; 0.02)	0.370	0.03 (0.01; 0.06)	**0.038**
Via Stoutness valorization	-0.05 (-0.09; -0.01)	**0.016**	0.19 (0.07; 0.32)	**0.002**
Via Dietary intake	0.17 (-0.12; 0.47)	0.246	-0.18 (-0.59; 0.23)	0.396
**Age**				
Via Intense physical activity	-0.01 (-0.01; 0.01)	0.611	0.01 (-0.01; 0.01)	0.251
Via Stoutness valorization	0.01 (0.01; 0.01)	**0.004**	0.02 (0.02; 0.03)	**0.000**
Via Dietary intake	0.02 (-0.03; 0.06)	0.480	0.03 (-0.01; 0.06)	0.132
**Urban duration**				
Via Intense physical activity	0.01 (-0.01; 0.01)	0.680	-0.01 (-0.01; 0.01)	0.135
Via Stoutness valorization	-0.01 (-0.01; 0.01)	0.169	-0.01 (-0.01; -0.01)	**0.045**
Via Dietary intake	0.01 (-0.02; 0.04)	0.427	-0.01 (-0.03; 0.02)	0.702

SES: Socioeconomic status, coded in an inverted direction.
