# Supplementary figures and images for: Direct and Indirect Determinants of Body Mass Index in Both Major Ethnic Groups Experiencing the Nutritional Transition in Cameroon
Source: Int J Environ Res Public Health. 2022 May 17;19(10):6108. doi: 10.3390/ijerph19106108 (PMC9141336; doi:10.3390/ijerph19106108)

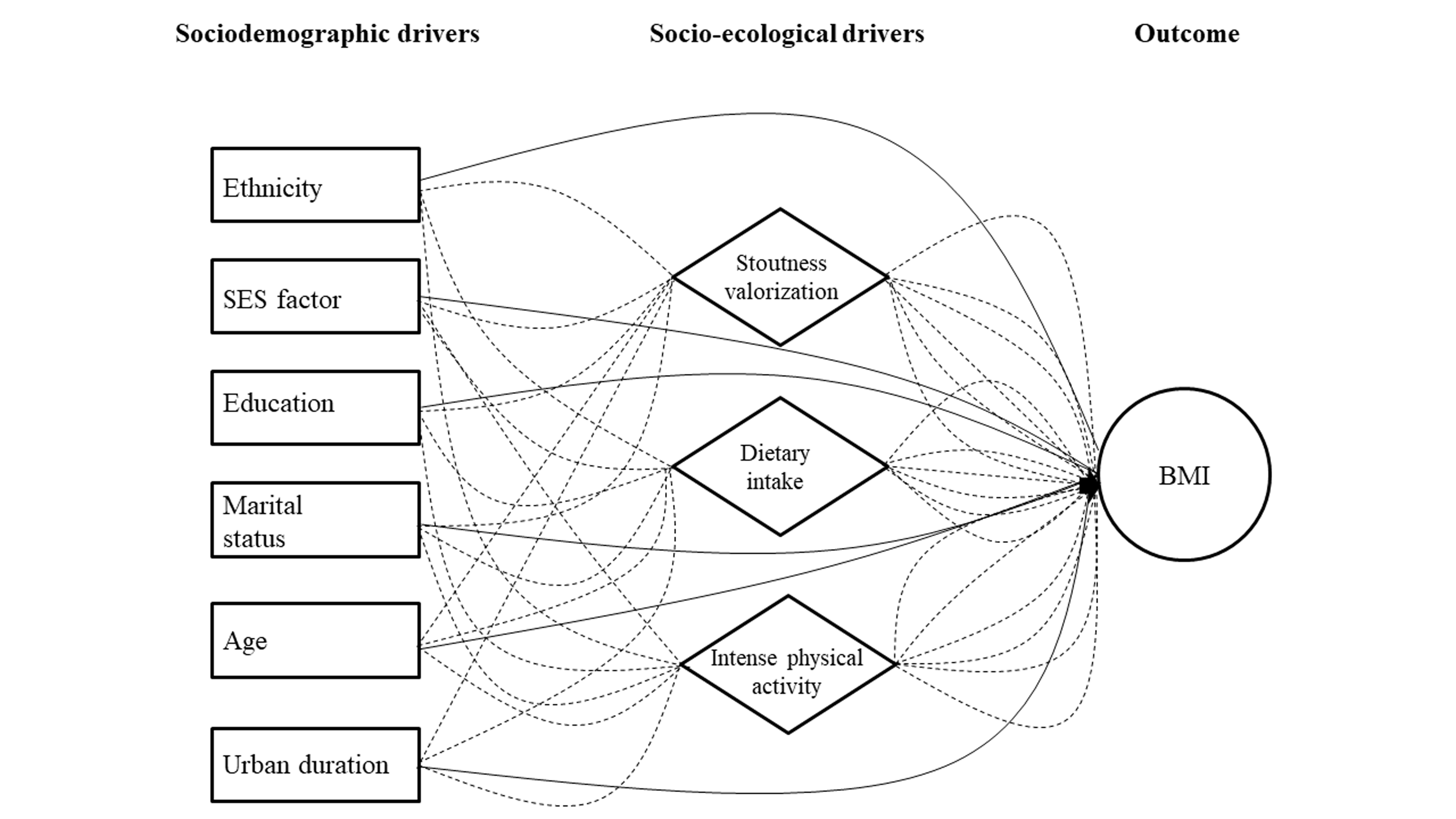

Supplement: Supplementary file 1 [file ijerph-19-06108-s001.zip › Figure S1.tif]
